# Microbiological Diversity and Associated Enzymatic Activities in Honey and Pollen from Stingless Bees from Northern Argentina

**DOI:** 10.3390/microorganisms12040711

**Published:** 2024-03-30

**Authors:** Virginia María Salomón, Johan Sebastian Hero, Andrés Hernán Morales, José Horacio Pisa, Luis María Maldonado, Nancy Vera, Rossana Elena Madrid, Cintia Mariana Romero

**Affiliations:** 1 Instituto Nacional de Tecnología Agropecuaria (INTA), Estación Experimental Agropecuaria Famaillá, PROAPI, Famaillá T4132, Argentina; salomon.virginia@inta.gob.ar (V.M.S.); maldonado.luismaria@inta.gob.ar (L.M.M.); 2 Planta Piloto de Procesos Industriales Microbiológicos (PROIMI-CONICET), Av. Belgrano y Pasaje Caseros, San Miguel de Tucumán T4001, Argentina; andymorales_2006@hotmail.com (A.H.M.); horacio_pisa@hotmail.com (J.H.P.); 3 Laboratorio de Medios e Interfases (LAMEIN), Departamento de Bioingeniería (DBI), Facultad de Ciencias Exactas y Tecnología (FACET), Universidad Nacional de Tucumán (UNT), Instituto Superior de Investigaciones Biológicas (INSIBIO-CONICET) DBI-FACET-UNT, INSIBIO-CONICET, Av. Independencia 1800, San Miguel de Tucumán T4001, Argentina; rmadrid@herrera.unt.edu.ar; 4 Facultad de Bioquímica, Química y Farmacia, Universidad Nacional de Tucumán (UNT), Chacabuco 461, San Miguel de Tucumán T4000, Argentina; veranr@gmail.com

**Keywords:** stingless bee, *Tetragonisca fiebrigi*, *Scaptotrigona jujuyensis*, honey, pollen, enzymatic activity

## Abstract

Honey and pollen from *Tetragonisca fiebrigi* and *Scaptotrigona jujuyensis*, stingless bees from northern Argentina, presented a particular microbiological profile and associated enzymatic activities. The cultured bacteria were mostly *Bacillus* spp. (44%) and *Escherichia* spp. (31%). The phylogenetic analysis showed a taxonomic distribution according to the type of bee that was similar in both species. Microbial enzymatic activities were studied using hierarchical clustering. *Bacillus* spp. was the main bacterium responsible for enzyme production. Isolates with xylanolytic activity mostly presented cellulolytic activity and, in fewer cases, lipolytic activity. Amylolytic activity was associated with proteolytic activity. None of the isolated strains produced multiple hydrolytic enzymes in substantial amounts, and bacteria were classified according to their primary hydrolytic activity. These findings add to the limited knowledge of microbiological diversity in honey and pollen from stingless bees and also provide a physiological perspective of this community to assess its biotechnological potential in the food industry.

## 1. Introduction

Stingless bees constitute a large taxa group (about 550 species) which includes the tribe Meliponini, which feature tiny stingers that cannot be used for protecting themselves [1]. They are found in most tropical and subtropical climes across the world and constitute the vast majority of native social bees in South America. They may be found throughout Argentina’s northern region, with the greatest diversity in the humid forests of the northeast [2].

Although knowledge of these bees is primarily based on cultural traditions and how native peoples employ them, formal studies in Argentina are relatively isolated and insufficient [3]. In this subject, formal meliponiculture is a relatively recent development. The use of man-made hives and their manipulation is becoming more popular in Argentina, but only a few species, like *Tetragonisca fiebrigi*, *T. aff. angustula*, and *Scaptotrigona jujuyensis*, are managed rationally. *Tetragonisca* hives are small and the harvest modest, but these bees are highly valued for the quality of their honey, while *Scaptotrigona* features a large hive size and a large honey harvest [2,4].

Stingless bee honey and pollen are prized for their medicinal and nutritional qualities. These bees collect and chemically change the floral nectars of native vegetation using their secretions. This mixture is stored and left to mature in cerumen pots or containers made of wax and resins, yielding unique honey with distinct properties when compared to *Apis mellifera* honey [5,6]. In this regard, one of the most noticeable changes is the higher percentage of humidity (usually greater than 20%), which makes it more fluid. This particularity causes fermentation processes driven by a specific microbial flora to occur during storage, influencing the composition, physicochemical qualities, and organoleptic characteristics [7]. Several researchers have noted that these kinds of honey have higher levels of acidity, moisture, and reducing sugars than *A. mellifera* honey, as well as varied sweetness, flavour, texture, scent, and therapeutic effects [8,9,10,11,12,13]. In this way, the honey and pollen generated by stingless bees create a unique physicochemical environment, giving them an appealing niche in the search for novel microbes and enzymes with high biotechnological value [14].

The main microorganisms found in honey are yeasts (*Penicillium* spp., *Mucor* spp., *Saccharomyces* spp., and *Schizosaccharomyces* spp.), which are responsible for its fermentation when the moisture content exceeds 21%. On the other hand, spore-forming bacteria, such as *Bacillus cereus* and *Clostridium* spp., are regularly found in *A. mellifera* honey [15]. The main microbial contamination comes from primary pre-harvest sources, which include pollen, the digestive tracts of bees, dust, air, and flowers [16]. Bees visit different environments, where they can acquire a wide community of microorganisms that contribute to nutrition, defence, and the acquisition of nutrients. Regarding secondary sources, we found handlers, equipment, or cross-contamination that occurred during the processing, manipulation, and transport of honey. Therefore, these processes must be controlled with good manufacturing practices. Among the microorganisms responsible for secondary contamination, there are different genera belonging to the Enterobacteriaceae family [17].

Although stingless bees share many similarities with *A. mellifera*, this group still hides many unexplored features [1]. It has been shown that specialised communities of bacteria reside in the guts of stingless bees [18]. In contrast to the Apini and Bombini tribes, *Lactobacillus* and *Bifidobacterium* genera are not always present or common in the Meliponini, Gilliamella, and Snodgrassella tribes. Moreover, stingless bees are reported to carry species of Acetobacter [19]. However, knowledge about their biodiversity is limited, because only a few species have reported on their intestinal bacterial microbiota [1]. Additionally, the majority of articles only discuss the roles of gut bacteria in feeding and defence against dangerous germs, but only a few investigations have focused on honey or pollen [20].

Based on the previously described literature, the objective of this work was to study the microbiological profiles of honey and pollen from two stingless bees native to northern Argentina, *Tetragonisca fiebrigi* and *Scaptotrigona jujuyensis*. Additionally, the enzymatic profiles of the cultured microorganisms were also assessed. In this approach, our work intends to make a substantial contribution to the research on the microbial diversity present in the honey and pollen of these species as well as its associated biotechnological potential.

## 2. Materials and Methods

### 2.1. Sampling of Honey and Pollen

Honey and pollen samples from *T. fiebrigi* and *S. jujuyensis* were collected from the Agricultural Experimental Station of INTA-Famaillá (Tucumán, Argentina). Aseptic sampling was performed from 9 hives in December 2016 and February 2017. A syringe was used to aspirate the honey, while the pollen was extracted using a sterile spatula. Samples were placed in sterile tubes and stored at −20 °C.

### 2.2. Isolation and Conservation of Microorganisms

For microorganism isolation, 2 g of honey and 1 g of pollen were dissolved in 18 mL and 9 mL of physiological 0.9% (*w*/*v*) NaCl solution, respectively. Successive dilutions were performed (100 μL) in different specific culture media: Man, Rogosa, and Sharpe (MRS), which is specific for *Lactobacilli* and lactic acid bacteria; Tryptic Soy Broth (TSB), a medium for spore-forming bacteria; and yeast extract, peptone, dextrose medium (YPD) for fungi and yeast. The plates were incubated aerobically at 30 °C for approximately 1–3 days. Single colonies were picked according to macroscopic characteristics (texture, colour, brightness, margins, and convexity) and re-streaked onto the solid medium previously used in each case. Microorganisms were incubated at 30 °C for 24 h to check the isolation purity, and then conservation was carried out in 20% glycerol at −80 °C [17].

### 2.3. Microbiological Quality

The microbiological quality was determined using the following analyses: (i) count of total mesophilic aerobic microorganisms; (ii) determination of total coliforms/g ISO 4831:2006 [21]; (iii) count of fungi and yeasts CFU/g ISO 21527:2008 [22]; and (iv) determination of sulphite reducers APHA 1992 ISO [23]. Experiments were performed in triplicate and data were statistically analysed according to Tukey’s tests (*p* < 0.05).

### 2.4. DNA Isolation and 16S rRNA Gene Amplification of Microorganisms

The microorganisms isolated had their genomic DNA extracted using the cetyltrimethylammonium bromide (CTAB) method [2]. Genomic samples were used to amplify the 16S rRNA genes using universal primers 27F and 1492R [24]. The reaction was run in a Mastercycler Gradient (Eppendorf, Hamburg, Germany) using the following cycle: 94 °C 4 min, 35 cycles of 1.5 min at 94 °C, 1.5 min at 55 °C, 2.0 min at 72 °C, and a final extension of 7 min at 72 °C. DNA quality and integrity were analysed by submerged electrophoresis in 0.8% (*w*/*v*) agarose gels stained with Gel Red^TM^ (Biotium) [25].

The products were purified with the Wizard SV Gel kit and PCR Clean-Up System (Promega Corp., Madison, WI, USA), and automated sequencing was performed by the Macrogen Facility (Macrogen, Seoul, Republic of Korea) [26]. The obtained reads were individually edited and entered online in the EZ taxon server [27] and through the Basic Alignment Search Tool (BLAST) (http://blast.ncbi.nlm.nih.gov/ accessed on 20 October 2023) to identify the most-related genera.

The 16S rRNA sequences were deposited in the GenBank database (https://www.ncbi.nlm.nih.gov/genbank/ accessed on 20 October 2023) for open access, reference code: OR021733-OR021769.

### 2.5. Phylogenetic Analysis

All 16S rRNA sequences obtained from the isolated microorganisms were aligned using the SINA service from the SILVA database [28] and edited to remove gaps and ambiguous nucleotides. Evolutionary distances were calculated using the Kimura 2-parameter method, and evolutionary history was inferred by employing the neighbour-joining (NJ) method using MEGA 11 [29]. Confidence values of the branches of the trees were determined by using bootstrap analyses based on 100 re-samplings.

### 2.6. Semiquantitative Determination of Enzymatic Activities

Enzymatic activities of the isolates were determined using differential media. The base culture medium used contained the following in g/L: yeast extract: 0.25, tryptone: 0.50, sodium chloride: 0.50, and agar: 15. In the case of proteolytic activity, 10 g/L of powdered milk (La Serenísima, General Rodríguez, Argentina) was added. For cellulolytic and xylanolytic activity, the medium was supplemented with 10 g/L carboxymethylcellulose (CMC) or beechwood xylan (Sigma Aldrich, Darmstadt, Germany), respectively. To determine amylolytic activity, 10 g/L soluble starch (Sigma Aldrich, Germany) was used as a substrate. Finally, to determine lipolytic activity, 2 g/L olive oil (AGD Co., General Deheza, Argentina) and rhodamine B (Sigma Aldrich, Darmstadt, Germany) were supplemented. The plates were incubated at 30 °C for 36 h.

The semiquantitative evaluation of enzymatic activities was carried out by measuring the diameters of the colonies and the halos produced by enzymatic degradation, using a Vernier caliper of 0–150 mm (with 0.1 mm subdivisions) (Merck, Darmstadt, Germany). Experiments were performed in triplicate.

Hydrolysis halos were revealed according to the type of enzymatic activity evaluated: (i) Proteolytic activity: the halo of degradation was observed as a clear zone around the colonies. (ii) Cellulolytic and xylanolytic activity: after microbial growth, a solution of 0.1% Congo Red (Sigma Aldrich) (*w*/*v* in distilled water) was added to the plates. It was rested for 15 min at room temperature and washed with a 0.1 M NaCl solution. (iii) Amylolytic activity: plates were covered with iodine solution, (0.3% (*p*/*v*) iodine and 1.0% (*p*/*v*) KI). Then, amylase-positive isolates were identified by the appearance of a clear zone around bacterial growth. (iv) Lipolytic activity: it was determined as a zone of fluorescence around the colonies by irradiation at 350 nm [2].

### 2.7. Hierarchical Analysis of Enzyme Activities of Isolated Microorganisms

For global analysis of the enzymatic activities of the isolated microorganisms, hierarchical grouping studies were carried out using Instant Clue V0.11.1 software. Halo/colony measurements were normalised based on the highest value recorded for each activity. Hierarchical clustering of isolates (rows) and enzyme activities (columns) was performed with the HCluster function, with a Euclidean distance metric and a linkage criterion of maximum or full-linkage clustering [30]. The combined results of both studies were presented as a heat map.

### 2.8. Statistical Analysis

All the experiments were performed in triplicate, and results were reported as the arithmetic means with its corresponding standard deviations. Tukey’s tests were performed using Minitab^®^ 17.1.0 statistical software (Minitab Inc., State College, PA, USA), and *p*-values <0.05 were considered statistically significant.

## 3. Results and Discussion

### 3.1. Microbiological Quality Analysis of Honey and Pollen

Since the moisture content of honey from stingless bees is higher than that of *A. mellifera*, it tends to be more susceptible to external contamination. For this reason, the microbiological quality analysis of these food products is critical [1].

The number of total mesophiles in the honey of both species varied considerably during the evaluated period (Table 1). When comparing the microbial counts in the honeys of both species, there were no significant differences (*p* < 0.05) in December 2016, while *T. fiebrigi* recorded a significantly larger number of total mesophiles in February 2017. On the other hand, both samples showed substantial differences (*p* < 0.05) in the counts for the total number of fungi and yeasts present in the honey: *S. jujuyensis* displayed a higher quantity of these microorganisms in December 2016, whereas *T. fiebrigi* did in February 2017. These differences depending on the harvest time were reported by other authors for honey from stingless bees, who concluded that the sampling period or season is related to the presence or absence of fungi and yeasts and mesophilic aerobes [31]. Furthermore, total mesophilic values were within the limits established in the European Standard for *A. mellifera* honey (<1 × 10^4^ CFU/g of honey), and the count of fungi were yeasts are within the values allowed in the Argentine Food Code (CAA) (10^3^–10^4^ CFU/g of honey). The reported values in this work agree with those in the literature, particularly for *Tetragonisca* spp. [17].

The count of mesophilic microorganisms in the pollen samples was significantly higher in *T. fiebrigi* than in *S. jujuyensis* in both years, while fungi and yeast values did not show significant differences between the samples. Since one source of pre-harvest contamination in honey includes pollen [16], the high values in the count of microorganisms related to *T. fiebrigi* honey during the second harvest (February 2017) (Table 1) may be connected with the content present in the pollen. All the values observed were found to be within the reference values in the CAA for *A. mellifera* pollen and were consistent with those reported by other authors [32].

Sulphite-reducing bacteria (*Clostridium* spp.) and those from coliform (*Salmonella* spp. and *Shigella* spp.)-related bacteria are quality indicators for honey since they signal food contamination and potential food poisoning. These microbes were not found in any of the honey or pollen samples from *T. fiebrigi* or *S. jujuyensis* during the sampling period, indicating that these honeys can be considered safe for consumption. In agreement with our results, [33] did not report the presence of coliforms in honeys of the genera *Frieseomelita*, *Nannotrigona*, *Partamona*, *Scaptotrigona,* and *Tetragonisca* that were collected in the state of Bahía, Brazil. The authors of [17] discovered that 42.85% of 28 *Tetragonisca angustula* hives tested positive for *Clostridium* and 39% for *Bacillus* spp., and they were negative for *Escherichia coli* and *Salmonella* spp. Coliforms in honey have been linked to contamination during processing, such as through the use of incorrect equipment and/or clothing [34]. As a result, it is deemed critical to establish honey extraction techniques and work on technical hive designs to prevent and/or mitigate contamination.

### 3.2. Microorganism DNA Isolation from Honey and Pollen of Stingless Bees

**Figure 1:**
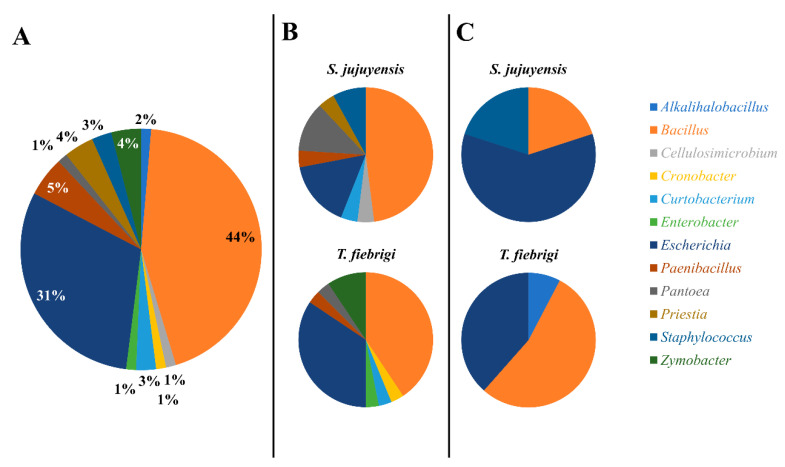
Microbial community diversity of the strains isolated in this study. (**A**) A total of 75 isolates were in all samples tested. Diversity of samples from (**B**) honey and (**C**) pollen, also differentiated by the species of the producing stingless bee.

A total of 75 microorganisms were isolated and sequenced, 57 isolates from honey (32 from *T. fiebrigi* and 25 from *S. jujuyensis*) and 18 isolates from pollen (13 from *T. fiebrigi* and 5 from *S. jujuyensis*). Molecular identification of the isolates was carried out through the amplification of a fragment of the 16S rRNA gene (Supplementary Data S1). Considering all samples regardless of their origin and species, it was possible to observe that two main genera were identified: *Bacillus* and *Escherichia*, with abundances of 44% and 31%, respectively (Figure 1A). When analysing the microorganisms identified by origin (honey or pollen) and species (*T. fiebrigi* or *S. jujuyensis*), a greater diversity was observed in the honey samples (Figure 1B) than those collected from pollen (Figure 1C). This could be because of the biotransformation conditions of mature bee bread to dry bee pollen, with changes in its pH and the production of antimicrobial compounds, which would affect the microbial diversity of the food product [35].

It was seen that *Bacillus* spp. dominated the isolates found in the honey of both communities of these stingless bees, accounting for 48% and 41% of all isolates for *S. jujuyensis* and *T. fiebrigi*, respectively. In both communities, *Escherichia* ranked as the second most diverse genus (16% and 35% for *S. jujuyensis* and *T. fiebrigi*, respectively). Lastly, several taxa, like *Priestia* and *Pantoea*, were only found in the honey made by *S. jujuyensis*; *Zymobacter*, *Cronobacter*, and *Enterobacter*, on the other hand, were only detected in the samples from *T. fiebrigi* (Figure 1B). Significant alterations in the diversity of the pollen samples from the two bee species were noted. In the pollen samples generated by *S. jujuyensis*, *Escherichia*-genus species were found to be completely dominant (60%), whereas *Bacillus* species predominated (54%) in the samples from *T. fiebrigi*. Another distinction was that, again, only the *S. jujuyensis* samples showed the presence of *Priestia* (20%) (Figure 1C).

The *Bacillus* genus predominance among the isolates agreed with other authors, who identified several species of *Bacillus* spp. in stingless bee species, such as *Heterotrigona itama* and *Melipona subnitida* [36,37]. Other authors also isolated *Bacillus* spp. in honey samples from *A. mellifera* [38].

A smaller abundance (<25%) of other taxa was also identified, such as *Staphylococcus*, *Enterobacter*, and *Pantoea*, which were previously reported in stingless bees [17]. In this regard, microbes belonging to the Pseudomonadota phylum were also discovered in honey bees [39]. This similarity of microbial communities between honey bees and stingless bees might reflect the similar roles in the colonies.

The Actinomycetota phylum was present only in the honey samples. These microorganisms were previously reported to be associated with the intestine of *A. mellifera jemenitica* in a proportion of 12% of the total bacteria identified [39]. Actinomycetota is associated with the production of compounds of an antimicrobial nature against potential pathogens of stingless bees [40]. Other authors proposed a model for *T. angustula*, in which actinobacteria are captured and transferred from the environment by bees, acting as vectors for these potentially beneficial microorganisms [4].

**Figure 2:**
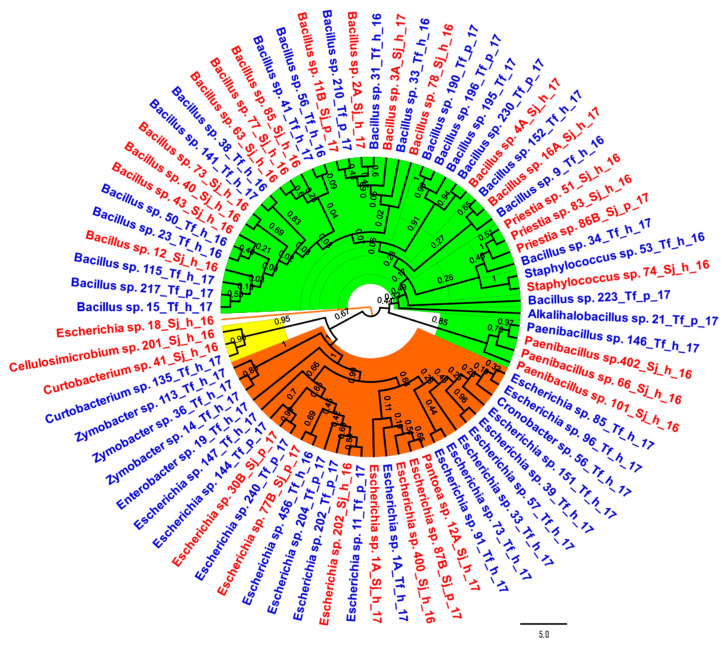
Phylogenetic tree was built by the neighbour-joining method based on 16S rRNA sequences of the 75 isolated strains. Strain names in blue corresponded to isolates from *Tetragonisca fiebrigi* samples; on the other hand, the strain names in red were from *Scaptotrigona jujuyensis*. Bacillota strains are highlighted in green, whereas isolates from the Pseudomonadota phylum are highlighted in orange, and strains from the Actinomycetota phylum are highlighted in yellow. Bar 5 substitutions per nucleotide position. All ambiguous positions were removed for each sequence pair (pairwise deletion option). There was a total of 3086 positions in the final dataset.

Regarding the phylogenetic analysis, the 75 sequences were grouped, as expected, into three large monophyletic groups: the phyla Bacillota (with a specific clade of *Paenibacillus* spp.), Pseudomonadota (with a clear differentiation from *Zymobacter* spp.), and Actinomycetota (Figure 2). Isolate 18_Sj_h_16, identified as *Escherichia* sp., presented the greatest evolutionary distance among the microorganisms evaluated.

When the taxonomic distribution of the samples was examined by bee species, the majority of the recognised genera were found in both kinds of stingless bees. Nevertheless, taxa such as *Alkalihalobacillus*, *Cronobacter*, *Enterobacter*, and *Zymobacter* were only discovered in *T. fiebrigi*. On the other hand, *Cellulosimicrobium* sp., *Pantoea* sp., and *Priestia* sp. were exclusively found in *S. jujuyensis* (Figure 2). This could be a consequence of bee species-specific traits, such as different foraging behaviours. It can be concluded that the trophic resources of both species of bees were different, changing the particular microbiota of their intestines. This could have caused taxonomically close species to be identified in the products of a bee species, decreasing the evolutionary distance of the isolates identified in honey and pollen when analysed based on the producing stingless bee.

### 3.3. Enzymatic Activities of Isolated Microorganisms

The integral study of the enzymatic activities present in the microorganisms was carried out by hierarchical clustering analysis. Isolates were grouped according to the enzymatic activity presented and their normalised halo/colony value. Of the 75 total isolates, 57 presented at least one type of activity studied, so they could be incorporated into the hierarchical analysis. Regarding the diversity of this subgroup, they did not present notable changes in their taxa distribution, almost totally conserving the structure of *Bacillus* and *Escherichia*, with a combined abundance of 79% (Supplementary Data S2).

**Figure 3:**
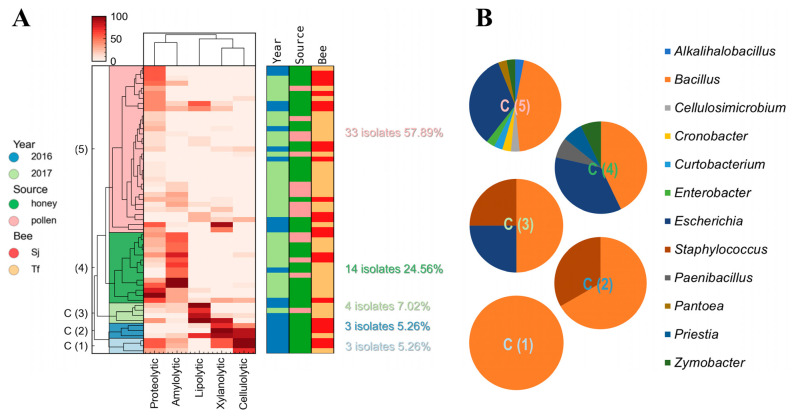
Enzyme-production study of the isolated strains in samples of honey and pollen from stingless bees from northern Argentina. (**A**) Heat map from the hierarchical clustering analysis of the strains that presented at least one of the types of enzyme activity evaluated (57 isolates). Values from 0 to 100 correspond to normalised enzyme activity data in all cases. Additional features such as harvest year, sample source, and producer bee type (Sj: *Scaptotrigona jujuyensis*, Tf: *Tetragonisca fiebrigi*) also helped to differentiate the examined strains, as indicated in the graphs to the right of the heat map. (**B**) Microbial diversity of the clusters formed by the hierarchical clustering method based on the enzymatic activities observed in the identified isolates.

As can be seen in the dendrogram of enzymatic activities, the microorganisms that had the highest values of xylanolytic activity (>50%) were more frequently associated with high cellulolytic activity (70–100%), and they exhibited lipolytic activity to a lesser extent (Figure 3A and Supplementary Data S3). It has been reported that both xylanases and cellulases can be induced by the same substrates, resulting in the simultaneous expression of both proteins, maximizing degradation and assimilation in the complex lignocellulosic biomass [41]. This has also been reported in cases of microbial communities, either in autochthonous niches or in defined co-cultures [42]. On the other hand, the isolates that presented the highest values of amylolytic activity were associated with the highest values of proteolytic activity, but in general, they did not present another type of enzymatic activity (Figure 3A).

**Figure 4:**
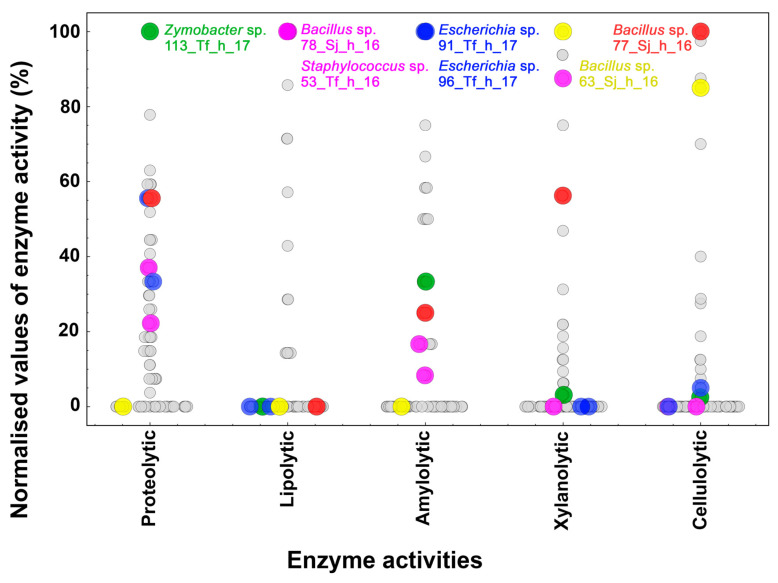
Swarm plot of the normalised values of the enzymatic activities present in the isolates. According to the activity under investigation, the distributions of each strain are shown (*x*-axis). The isolates with the highest values (100%) of proteolytic (green), lipolytic (pink), amylolytic (blue), xylanolytic (yellow), and cellulolytic (red) activity are highlighted.

The microorganisms evaluated (57 strains) were grouped into 5 clusters, with cluster 1 (C1) and cluster 2 (C2) each composed of 3 isolates (5.26%); cluster 3 (C3) of 4 (7.02%); cluster 4 (C4) of 14 (24.56%); and finally, cluster 5 (C5) of 33 isolates (57.89%) (Figure 3A). C1 and C2 presented the highest values of xylanolytic and cellulolytic activity, while the main producers of lipolytic enzymes were grouped in C3. C4 had the highest amylolytic activity and was grouped with microorganisms with a proteolytic capacity with little or no activity against CMC and xylan. Finally, C5 made up of most of the isolates and was grouped with microorganisms with low catalytic performance, mainly protease producers (Figure 3A). Through this hierarchical grouping, a large part of the microorganisms that made up the communities under study were not hydrolytic enzyme producers, or were scarcely producers, under the conditions assessed (growth times, solid culture media, and substrates). However, among all the isolated microorganisms, several *Bacillus* strains, such as 78_Sj_h_16, 63_Sj_h_16, and 77_Sj_h_16, showed intriguing capabilities as enzyme producers. These strains had the best values of lipolytic, xylanolytic, and cellulolytic activity, respectively (Figure 4). These enzymes, which were present in honey and pollen, could have an impact on the characteristics of bee food products [43].

The conformation of these clusters was analysed according to (i) the year of collection, (ii) the material collected, and (iii) the species of bee (Supplementary Data S4). C1 and C2 comprised microorganisms isolated in honey, a large part of which were found in *S. jujuyensis* samples during the December 2016 collection. However, it is important to note that as we move from C1 to C5, we can see three generalised trends: as the number of isolates collected in February 2017 increased, more bacteria were isolated from *T. fiebrigi* samples, and the number of strains that displayed interesting enzymatic activity values (greater than 50% of the maximum activity observed in all the isolates) decreased (Figure 3A). Thus, C5 comprised 69.70% of the isolates from February 2017 and 60.61% from *T. fiebrigi*. However, when analysing the conformation of C3, which was made up of the microorganisms with the highest values of lipolytic activity, the microorganisms were mostly isolated from *T. fiebrigi* (75.00%), but the majority were collected in December 2016 (75.00%) (Supplementary Data S4). The difference between the December 2016 and February 2017 isolates could be because of the climatic conditions that affected the foraging of the different bee species and, therefore, their microbial composition.

Regarding the clusters’ microbiological diversity, they varied markedly (Figure 3B). C1 was only formed of different species of the genus *Bacillus*. C2 also presented mostly *Bacillus* (66.67%) and *Staphylococcus* (33.33%). In addition to these two genera, C3 included *Escherichia* (25.00%). C4 was mainly a compound of *Bacillus* spp. (42.86%) and *Escherichia* spp. (35.71%), in addition to other species in minimal abundance: *Paenibacillus* spp. (7.14%), *Priestia* spp. (7.14%), and *Zymobacter* spp. (7.14%). Finally, C5 was the most diverse, presenting the following genera: *Bacillus* (44.00%), *Escherichia* (30.67%), *Alkalihalobacillus*, *Cellulosimicrobium*, *Cronobacter*, *Curtobacterium*, *Enterobacter*, *Paenibacillus*, *Pantoea*, *Priestia*, *Staphylococcus*, and *Zymobacter* (Figure 3B). Analysing this microbiological diversity and the enzymatic activities present in the different clusters, it can be concluded that those with higher values of enzymatic activity (C1, C2, and C3) were dominated by *Bacillus* spp. On the other hand, as catalytic activity values decreased in C4 and C5, their microbial compositions varied significantly, increasing the importance of the genus *Escherichia* and others belonging to the Pseudomonadota taxon, such as *Cronobacter*, *Enterobacter*, *Pantoea*, and *Zymobacter*.

*Bacillus* spp. microorganisms were primarily responsible for enzyme production in the different communities evaluated. Also, *Bacillus* species could play a role both in the metabolic conversion of food by bees and in the control of microbes that compete or lead to deterioration [35]. This interspecific relationship between *Bacillus* spp. and the rest of the community was observed in both other food isolates and in cultures defined in the laboratory [44]. This explains the abundance of these species that was found in samples isolated in this work. In particular, it has been postulated that worker stingless bees can inoculate *Bacillus* spp. in the food they provide for their larvae and that these microorganisms could predigest, convert, and/or preserve food components [35].

**Figure 5:**
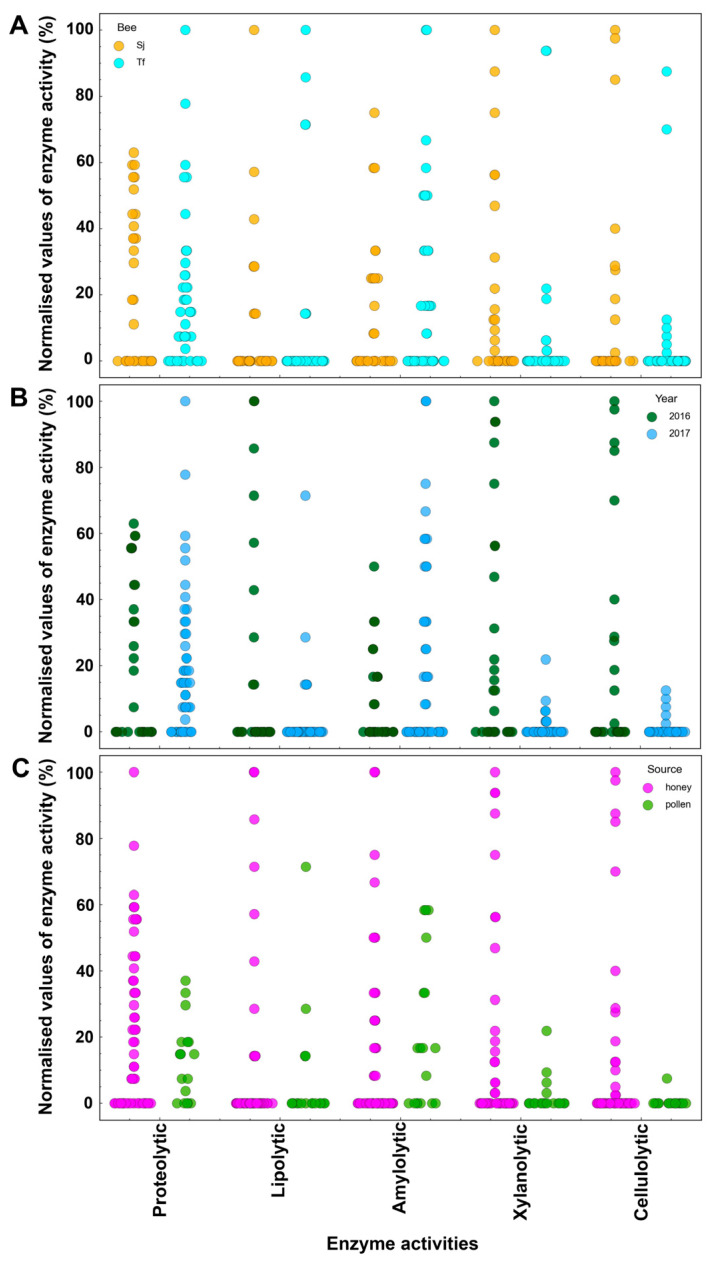
Swarm plots of the normalised enzymatic activity levels displayed by the strains, dispersed based on the traits of the samples in which the microorganisms were isolated: bee species (**A**), harvest year (**B**), and type of sample (**C**). The enzymatic activities under study are depicted on the *x*-axis, and the strains’ varying levels of each activity are shown.

Swarm plots were made to analyse the enzymatic production and the best-producing microorganisms based on the data of the normalised activities (Figure 4 and Figure 5). The highest level of proteolytic activity was found in an isolate of *Zymobacter* sp., 113_Tf_h_17 (Figure 4, green spots), whereas for the production of lipolytic enzymes, the isolates identified as Bacillota, *Staphylococcus* sp. (53_Tf_h_16), and *Bacillus* sp. (78_Sj_h_16) presented the greatest degradation values (Figure 4, pink spots). In terms of xylanolytic and cellulolytic activities, two strains of *Bacillus* spp. (63_Sj_h_16 and 77_Sj_h_16, respectively) exhibited the greatest potential for polysaccharide hydrolysis (Figure 4, yellow and red spots, respectively). These strains of *Bacillus* spp. were isolated in the same ecological niche, honey produced by *S. jujuyensis*, during the December 2016 harvest, which indicates favourable conditions for microorganisms capable of hydrolysing lignocellulose. In this sense, there is evidence that honey from stingless bees can ferment naturally inside sealed pots. This type of fermentation is carried out by *Bacillus* spp. [45], which is consistent with the various hydrolytic activities reported in this work. Finally, the best values of amylolytic activity were reported in two isolates from the same niche, 91_Tf_h_17 and 96_Tf_h_17, both of which were identified as *Escherichia* spp. (Figure 4, blue spots). The metabolism of starch degradation by species of this genus is well documented [46].

There were no isolated microorganisms with important productions of various enzymatic activities. In most of the evaluated substrates, when an isolate presented high values of catalytic activity, at the same time, it showed poor or no activity against other polymers, except for xylanolytic microorganisms, which present high cellulolytic activity [41]. Based on these results, there was not an enzymatic “superproducer” microorganism in the samples analysed, but in the microbial community, there was a marked “enzymatic work specialization”, where the degradations of the different substrates were differentiated between species and even between genera.

When analysing the swarm plots in terms of the year, material collected, and bee species, it can be observed that according to the bee species, no significant differences were recorded in the isolation of enzyme-producing microorganisms. The most noticeable difference was observed in terms of proteolytic activity, where *T. fiebrigi* had a greater number of isolates (Figure 5A). Evaluating the year of collection, a clear distinction was observed. Proteolytic and amylolytic activities showed higher values in the February 2017 isolates, which correspond to C4 and C5. In contrast, the rest of the activities (lipolytic, xylanolytic, and cellulolytic) were higher in strains isolated in December 2016 (Figure 5B). Because of these results and the hierarchical clustering analysis, the year of the collection was the most influential factor in determining the predominance of one type of enzymatic activity over the others in the microbial communities, possibly because of climatic conditions during the stages of foraging and food production in the hives. Finally, the distribution of the producing microorganisms was evaluated depending on the source of food, honey, or pollen (Figure 5C). Despite there being fewer isolates obtained from pollen, an interesting trend can be observed depending on the type of enzymatic activity evaluated. In all cases, the microorganisms reported low values of enzymatic activity (<60%), except for the 202_Tf_p_17 strain, with a percentage of lipolytic activity of 71.42%. This behaviour was greater when the distribution was studied regarding activities against xylan and CMC, with maximum activity values of 21.85% and 7.50%, respectively, being reported. This distribution could be because pollen is the main source of lipids and other components (proteins and vitamins) necessary for the development of bee colonies [47]. It means that this food would favour the growth and proliferation of fatty acid-degrading microorganisms.

Although this study represents one of the first approaches to microbial diversity and its associated enzymatic potential in honey and pollen from stingless bees (particularly *T. fiebrigi* and *S. jujuyensis*), we consider that one of the main limitations of our work lies in that many of the microorganisms present in the studied matrices may not be culturable under the conditions tested. This means that the biotechnological potential observed in our work could be underestimated. An interesting approach to complement our results would be to use omics techniques (metagenomics, proteomics, transcriptomics, etc.) to corroborate and expand on the information, as opposed to the traditional approach for studying enzymatic bioprospecting, which requires defined culture media [48,49]. By thoroughly investigating the microbial diversity in honey and pollen, we might be able to uncover new compounds with antibacterial action and potentially probiotic microorganisms, among other things, broadening the scope of possible biotechnological uses. In this regard, one of the bacterial isolates from *S. jujuyensis* honey has shown the capacity to produce a biopolymer with prebiotic characteristics in addition to probiotic potential [2].

## 4. Conclusions

In conclusion, this two-year study examined the microbial presence in two Meliponini stingless bees, *Tetragonisca fiebrigi* and *Scaptotrigona jujuyensis*. Regarding the year of harvest, it has been observed that the counts of fungi and yeasts varied depending on the bee species. The total mesophilic counts of *T. fiebrigi* honey, on the other hand, showed changes depending on the year of collection. *Bacillus* spp. and *Escherichia* spp. were the two most common microorganism genera found in the honey and pollen. In this respect, *Bacillus* spp. was in charge of producing the majority of the enzymes. This has effects on the well-being of colonies and the quality of their outputs, such as honey. Moreover, controlled studies with stingless bee colonies housed in one place and under continuous monitoring are needed to ascertain whether seasonal changes occur. This study adds to the body of knowledge concerning the microbiology of honey and pollen from stingless honey bees, as well as highlighting the biotechnological potential of these products based on their enzymatic profiles.

## Figures and Tables

**Table 1:** Microbiological analysis in honeys and pollen from *Tetragonisca fiebrigi* and *Scaptotrigona jujuyensis*. The results are expressed as CFU/g of the sample. Analysis of comparison of means according to Tukey’s test. Different letters indicate significant differences between the values of the same year and different species of bees (*p* < 0.05).

Assay	Bee	Honey		Pollen	
		December 2016	February 2017	December 2016	February 2017
Total Mesophilic Microorganisms	*T. fiebrigi*	6.2 × 10^1^ ± 3.5 ^(A)^	3.0 × 10^3^ ± 2.6 × 10^2 (A)^	4.5 × 10^1^ ± 3.7 ^(A)^	4.4 × 10^2^ ± 1.5 × 10^1 (A)^
	*S. jujuyensis*	6.4 × 10^1^ ± 1.4 × 10^1 (A)^	3.0 × 10^1^ ± 0.7 ^(B)^	3.2 × 10^1^ ± 5.4 ^(B)^	3.5 × 10^1^ ± 2.0 ^(B)^
Fungi and Yeasts	*T. fiebrigi*	3.8 × 10^1^ ± 1.2 ^(B)^	4.0 × 10^3^ ± 3.0 × 10^2 (A)^	2.2 × 10^1^ ± 0.8 ^(A)^	4.6 × 10^1^ ± 3.7 ^(A)^
	*S. jujuyensis*	8.9 × 10^2^ ± 9.7 ^(A)^	2.0 × 10^1^ ± 0.6 ^(B)^	1.8 × 10^1^ ± 6.4 ^(A)^	2.4 × 10^1^ ± 3.2 ^(A)^

## Data Availability

The 16S rRNA sequences acquired from the isolates investigated in this study have been submitted in the GenBank database (https://www.ncbi.nlm.nih.gov/genbank/), for open access, reference code: OR021733-OR021769.

## Supplementary Materials

The following supporting information can be downloaded at: https://www.mdpi.com/article/10.3390/microorganisms12040711/s1. Supplementary Data S1 (spreadsheet): Names of the isolates and results of the identification through the Basic Alignment Search Tool of the 16S rRNA gene. The microorganisms’ closest hit accession numbers are from the National Center for Biotechnology Information (NCBI) database. Supplementary Data S2 (Figure): Microbial community diversity of the subgroup formed by strains isolated and identified from honey and pollen that presented at least one of the types of enzyme activity evaluated. Supplementary Data S3 (spreadsheet): Hierarchical cluster analysis of the enzymatic production of microbial isolates. Enzymatic activity values are normalised and expressed as percentages. Isolate diversity, as well as honey and pollen sample characteristics (harvest year, sample type, and producer bee—Sj: *Scaptotrigona jujuyensis*, Tf: *Tetragonisca fiebrigi*) were also analysed. Supplementary Data S4 (Table): Conformation of the clusters was established by hierarchical clustering analysis according to sample features (year of collection, type of material, and bee species) from the strains with enzymatic activity which were isolated.
