# Supplementary material for: Microbiological Diversity and Associated Enzymatic Activities in Honey and Pollen from Stingless Bees from Northern Argentina
Source: Microorganisms. 2024 Mar 30;12(4):711. doi: 10.3390/microorganisms12040711 (PMC11051704; doi:10.3390/microorganisms12040711)
Supplement: Supplementary file 1 [file microorganisms-12-00711-s001.zip › Supplementary Data.pdf]

Supplementary Data

Supplementary Data S1. Name of the isolates and results of the identification through the Basic Alignment Search Tool of 16S rRNA gene. The microorganism closest hit accession numbers are from the National Center for Biotechnology Information (NCBI) database.

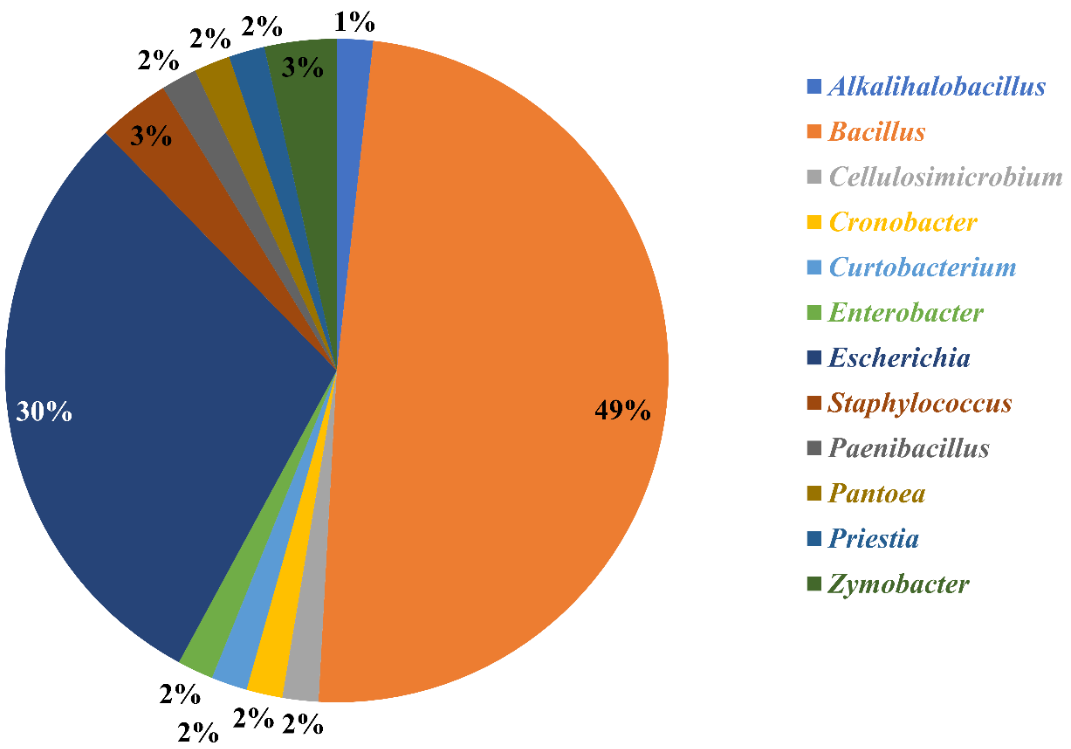

Supplementary Data S2. Microbial community diversity of the subgroup formed by isolated and identified strains from honey and pollen, that presented at least one type of enzyme activity evaluated.

Supplementary Data S3. Hierarchical cluster analysis of the enzymatic production of microbial isolates. Enzymatic activity values are normalized and expressed as a percentage. Isolates diversity as well as honey and pollen samples characteristics (harvest year, sample type and producer bee - Sj: *Scaptotrigona jujuyensis*, Tf: *Tetragonisca fiebrigi* -) were also analyzed.

Supplementary Data S4. Conformation of the clusters established by hierarchical clustering analysis according to samples features (year of collection, the type of material and bee species) from which the strains with enzymatic activity were isolated.

Cluster ID	***Year***	***% of total***	***Source***	***% of total***	***Bee***	***% of total***
**C (1)**	2016	100.00	honey	100.00	*S. jujuyensis*	66.67
	2017	0.00	pollen	0.00	*T. fiebrigi*	33.33
**C (2)**	2016	100.00	honey	100.00	*S. jujuyensis*	66.67
	2017	0.00	pollen	0.00	*T. fiebrigi*	33.33
**C (3)**	2016	75.00	honey	75.00	*S. jujuyensis*	25.00
	2017	25.00	pollen	25.00	*T. fiebrigi*	75.00
**C (4)**	2016	14.29	honey	71.43	*S. jujuyensis*	28.57
	2017	85.71	pollen	28.57	*T. fiebrigi*	71.43
**C (5)**	2016	30.30	honey	66.67	*S. jujuyensis*	39.39
	2017	69.70	pollen	33.33	*T. fiebrigi*	60.61
